# Long-Term Evaluation of Breeding Scheme Alternatives for Endangered Honeybee Subspecies

**DOI:** 10.3390/insects11070404

**Published:** 2020-06-30

**Authors:** Manuel Plate, Richard Bernstein, Andreas Hoppe, Kaspar Bienefeld

**Affiliations:** 1 Institute for Bee Research, Friedrich-Engels Str. 32, 16540 Hohen Neuendorf, Germany; richard.bernstein@hu-berlin.de (R.B.); andreas.hoppe@hu-berlin.de (A.H.); kaspar.bienefeld@hu-berlin.de (K.B.); 2 Albrecht Daniel Thaer-Institute for Agricultural and Horticultural Sciences, Humboldt University of Berlin, 10099 Berlin, Germany

**Keywords:** honeybee breeding, endangered species, simulation studies, sustainable breeding, inbreeding, local subspecies, breeding strategies, genetic variance, genetic gain

## Abstract

Modern breeding structures are emerging for European honeybee populations. However, while genetic evaluations of honeybees are becoming increasingly well understood, little is known about how selection decisions shape the populations’ genetic structures. We performed simulations evaluating 100 different selection schemes, defined by selection rates for dams and sires, in populations of 200, 500, or 1000 colonies per year and considering four different quantitative traits, reflecting different genetic parameters and numbers of influential loci. Focusing on sustainability, we evaluated genetic progress over 100 years and related it to inbreeding developments. While all populations allowed for sustainable breeding with generational inbreeding rates below 1% per generation, optimal selection rates differed and sustainable selection was harder to achieve in smaller populations and for stronger negative correlations of maternal and direct effects in the selection trait. In small populations, a third or a fourth of all candidate queens should be selected as dams, whereas this number declined to a sixth for larger population sizes. Furthermore, our simulations indicated that, particularly in small populations, as many sires as possible should be provided. We conclude that carefully applied breeding provides good prospects for currently endangered honeybee subspecies, since sustainable genetic progress improves their attractiveness to beekeepers.

## 1. Introduction

Recent years have seen a notable increase in efforts to improve breeding strategies for the western honeybee (*Apis mellifera*) both theoretically [1,2,3] and practically [4,5,6,7]. As a consequence, for many of the European subspecies, organized breeding activities are being developed for the first time [8,9,10]. The newly established breeding populations vary greatly in size, ranging from populations with several thousand potential breeding queens per year, which is the case for the Carniolan bee (*A. m. carnica*), to highly endangered subspecies, such as the Maltese honeybee (*A. m. ruttneri*), with only a few hundred colonies left [10,11,12]. Many of the native subspecies in Europe are currently in danger of replacement or hybridization with economically more attractive subspecies such as *A. m. carnica* or *A. m. ligustica* [12]. The Asian honeybee (*Apis cerana*) is facing similar threats [13]. While breeding activities for this species are still scarce, it is widely assumed that it can be bred in the same way as *A. mellifera*, and the first breeding efforts yielded promising results [14,15].

Genetic improvement of local subspecies through breeding provides a great chance for their conservation, since keeping them becomes more attractive [16,17,18]. However, whenever breeding is practiced in small populations, the benefits of genetic improvement have to be weighed against the negative effects of increased inbreeding rates and loss of genetic variance [18,19,20,21]. The Food and Agriculture Organization of the United Nations (FAO) suggests that breeding schemes for agricultural species should not lead to an increase in inbreeding coefficients of more than 0.5% to 1% per generation [22]. This is equivalent to an effective population size of $N_{e}=50$ to $N_{e}=100$. These thresholds have recently been used to evaluate breeding populations in a wide range of domesticated species, including cattle [23], swine [24], sheep [25], chicken [26], horses [27], rabbits [28], dogs [29], and donkeys [30].

The development of inbreeding in a population can be steered by the selection rates for dams and sires. In most livestock species, selection rates are assessed by the (total or relative) number of selected parents [31,32]. However, in honeybee breeding, the intensity of selection for dam queens is commonly determined indirectly via the sizes of sister groups of queens. If the population size is constant, the notions of selection rate and sister group size are reciprocal: If a relative number *r* of queens are selected as dams, each dam will on average have 1/r offspring. A likely reason for this peculiar terminology in honeybee breeding is that, unlike in other species, breeders can easily regulate the number of queen offspring from a dam by grafting larvae [5].

In modern honeybee breeding, much pioneer work has been established by Brother Adam of Buckfast Abbey. Starting in the 1920s, he followed the approach of combining desirable properties of different subspecies by controlled hybridization. This required extensive performance tests, during which he placed numerous sister groups of colonies (i.e., colonies whose queens shared a common dam) on a variety of apiaries to obtain reliable results regarding which genetic lines proved superior. In his breeding practice, the sizes of these sister groups regularly exceeded the numbers of 20 or even 30 colonies [33]. Working with such big sister groups, however, implies that, assuming a constant population census, only 3% to 5% of all queens can be selected as dams. While this may be possible in crossbreeding schemes like those for Buckfast bees, such sharp selection can hardly be sustainable for breeding in closed populations. In 1972, the International Federation of Beekeepers’ Associations (Apimondia) recommended performance tests with sister group sizes of 12 queens; i.e., a selection rate of 8.3% [34]. Since then, the introduction of best linear unbiased prediction (BLUP) breeding value estimation in the 1990s has facilitated the genetic evaluation of queens by taking more remote relationships into consideration [35,36]. Some honeybee breeding associations have thus relaxed their recommendations to sister group sizes of six to eight queens (12.5% to 16.6% of queens selected as dams) [37,38], while other organizations still evaluate groups of 12 sister queens [5,39].

Not only the selection intensity on the dam’s side, but also the selection of sires has an influence on honeybee populations. However, the latter is complicated by the honeybee’s mating biology. Shortly after hatching, virgin queens perform one or several flights to nearby drone congregation areas where they mate with several drones from other colonies [40]. Currently, the most common ways to guarantee controlled mating of queens with valuable drone material are the use of isolated mating stations and artificial insemination [7]. On mating stations, where virgin queens are brought to perform their nuptial flights, drones are provided by colonies that are set up specifically for this purpose. By geographic remoteness, the presence of other drones can be excluded. Choosing the queens of the drone-producing colonies to share a common dam with superior breeding values achieves a high genetic standard of drones [1]. The average number of drones a queen mates with on a mating station is often assumed to be 12 [2,40,41,42]. Providing controlled mating for honeybees requires considerable organization and logistics, but as shown in a previous study [43], it is paramount for successful breeding, because otherwise the genetic gain is severely reduced. Drones are haploid and their only known purpose for a honeybee colony lies in their role during reproduction; hence, they are regularly referred to as *flying gametes* [1,44,45]. The collective of drone-producing queens on a mating station is seen as the analogue to *sires* in dioecious diploid animals [2,3,35]. The choice of how many mating stations are set up for a honeybee breeding population thus represents the absolute number of sires, and hence determines the sharpness of selection on the paternal path.

In the 1980s, computer simulations became a popular tool to evaluate honeybee breeding strategies. Moritz [46] evaluated different within-family and mass selection schemes for their implications on inbreeding and genetic progress, while Page and Laidlaw [47] introduced and examined systems of line breeding and outcrossing. However, these studies had to make vast simplifications in the relation of inbreeding and genetic progress and did not include modern techniques of genetic evaluation. In the last decades, Monte Carlo simulations for farm animals have grown more powerful in manifold respects, allowing for investigations of complex traits under modern breeding schemes on the level of individuals [48,49]. In an earlier study, we introduced the program BeeSim, which made these concepts available for the honeybee with its biological peculiarities [3]. Now, we use it to examine a great variety of breeding schemes to study the long-term implications of different selection intensities on honeybee breeding populations of different sizes.

## 2. Materials and Methods

### 2.1. Breeding Scheme

We used the program BeeSim [3] to examine breeding populations of three different sizes. The small population (S) comprised N=200, the medium population (M) comprised N=500, and the large population (L) comprised N=1000 breeding queens per year (see Table 1 for a summary of the used variables). Here, the term *breeding queens* is used in the sense of *selection candidates* and as a distinction from drone-producing queens on mating stations. For each of the populations S, M, and L, we ran simulations over the course of 100 years in which we selected for a single directly (worker group) and maternally (queen) influenced trait. In each year, a BLUP breeding value estimation was carried out based on simulated performance data, and the best two-year-old breeding queens were selected to produce the next generation of breeding queens. We simulated ten different selection rates on this path, represented by the number $k_{d}$ ($1\leq k_{d}\leq 10$) of queens that shared the same dam; i.e., $\left\lfloor N/k_{d}\right\rfloor$ dam queens were selected across families and each selected queen produced $k_{d}$ daughter queens. In cases where $k_{d}$ did not divide *N*, the remaining queens had dams that were randomly chosen among the selected two-year-old breeding queens.

All queens were brought to isolated mating stations where they mated with twelve drones each. The drones on a mating station came from eight drone-producing colonies, the queens of which shared a common dam (see [1,35] for more detailed descriptions of honeybee mating stations). We simulated ten different selection intensities on the sires’ path by different numbers of mating stations, relative to the total population; i.e., in each year, the $k_{s}$% ($1\leq k_{s}\leq 10$) best three-year-old breeding queens were selected to produce the set-up of one mating station each.

The different choices of $k_{d}$ and $k_{s}$ stood for different selection intensities on the maternal and paternal paths, respectively. In combination, they defined 100 distinct selection schemes for each of the populations S, M, and L. For given $k_{d}$ and $k_{s}$, we named the corresponding breeding scheme $S_{k_{s}}^{k_{d}}$.

To facilitate the interpretation of the simulation results, we give a general idea of how $k_{d}$ and $k_{s}$ influence genetic gain and inbreeding in quantitative genetic theory—particularly because the sister group size $k_{d}$ is a non-common variable in this context. Selection response depends linearly on the selection intensity *i*, which in turn can be calculated from the selection rates $k_{s}$ on the sire’s path and $1/k_{d}$ on the dam’s path. Note in particular that intensified selection corresponds to higher values of $k_{d}$ but lower values of $k_{s}$. Inbreeding rates depend on absolute numbers of selected dams and sires rather than selection rates. Making vast simplifications, Wright [50] estimated the inbreeding rate in a population with *M* reproducing males and *F* reproducing females as ΔF≈(F+M)/8MF. While this formula should not be directly applied to honeybees, it still signifies that inbreeding rates are mainly determined by the total numbers $N/k_{d}$ of dams and $N\cdot k_{s}$ of sires.

### 2.2. Genetic Models

The queens’ genetics were simulated based on a finite locus model with $N_{l}=200$ or $N_{l}=400$ unlinked loci and were inherited according to the Mendelian rules without mutations, as described in [3]. All selection traits had an initial maternal additive variance of $\sigma_{A,m}^{2}=1$, an initial direct additive variance of $\sigma_{A,d}^{2}=2$, and a residual variance of $\sigma_{E}^{2}=1$. Two different values were chosen for the covariance between maternal and direct effects: A moderately negative covariance between maternal and direct effects of $\sigma_{A,md}=-0.75$ and a strongly negative covariance of $\sigma_{A,md}=-1.25$ were considered. The resulting total genetic standard deviations in the sum of maternal and direct breeding values of queens (the inheritance criterion) were $\sigma_{A,\mathrm{IC}}=1.22$ and $\sigma_{A,\mathrm{IC}}=0.71$. The setting with moderate negative correlation ($\sigma_{A,md}=-0.75$) reflected initial maternal, direct, and total heritabilities of $h_{m}^{2}=0.53$, $h_{d}^{2}=0.34$, and $h^{2}=0.79$, respectively. The correlation between both effects was $r_{md}=-0.53$. The corresponding values for the traits with strong negative correlation ($\sigma_{A,md}=-1.25$) were $h_{m}^{2}=0.72$, $h_{d}^{2}=0.46$, $h^{2}=0.36$, and $r_{md}=-0.88$ (heritabilities calculated according to Brascamp and Bijma [42]). These parameters have previously been used in honeybee-specific breeding simulations [3,43] and are in accordance with what has been estimated for European honeybee populations for economically relevant traits, such as honey yield and gentleness [41,51]. The set-up of individual loci of the queens, workers, and drones from the base population as well as the inheritance of alleles to later generations were performed by the BeeSim software [3]. All loci were simulated as purely additive and biallelic, while allele frequencies followed a U-shaped β(0.5,0.5)-distribution. Allele effects were sampled following a mixture of multivariate Laplace and Normal distribution with variance $\Sigma_{A}=\left[\begin{array}{cc} \sigma_{A,m}^{2} & \sigma_{A,md}\\ \sigma_{A,md} & \sigma_{A,d}^{2} \end{array}\right]$ and post-corrected to yield the desired genetic variances exactly. This procedure was identical to that used in [3], where it is described in greater detail. The colonies’ performance records were generated as the sum of their queens’ maternal breeding values, their worker groups’ direct breeding values, and residual values with variance $\sigma_{E}^{2}$. All fixed environmental effects were modeled to be zero.

The different combinations of the numbers of loci ($N_{l}=200$ or $N_{l}=400$) and initial negative correlations of maternal and direct effects ($r_{md}=-0.53$ or $r_{md}=-0.88$) led to four distinct selection traits, which we refer to as $T_{200}^{-0.53}$, $T_{400}^{-0.53}$, $T_{200}^{-0.88}$, and $T_{400}^{-0.88}$, respectively.

For each of the 1200 combinations of population size (S, M, L), selection scheme ($S_{k_{s}}^{k_{d}}$, $1\leq k_{d},k_{s}\leq 10$) and trait ($T_{200}^{-0.53}$, $T_{400}^{-0.53}$, $T_{200}^{-0.88}$, $T_{400}^{-0.88}$), simulations were carried out with 24 repetitions in order to obtain stable results.

### 2.3. Breeding Value Estimation

Based on the simulated performance tests, we carried out a BLUP breeding value estimation using the BLUPF90 software [52]. The inverse numerator relationship matrix was calculated with a bee specific approach, as it is described by Bernstein et al. [2], following the ideas of Brascamp and Bijma [1]. Each of the simulated colonies was assigned to one of 7N/100 apiaries, leading to an average apiary size of 14.3 colonies. In the breeding value estimation, each combination of year and apiary was considered as a fixed effect. The queens of the base population were assigned to the apiaries randomly. In later generations, 70% of queens were assigned the same apiary as their dam, while the remainder were assigned to random apiaries. This steadiness in apiaries resembles that of the Central European breeding population represented by beebreed.eu [53].

In the first years of selection, the previously defined exact genetic parameters were used as inputs for the breeding value estimation. However, in the finite locus model, the allele frequencies within a population under selection shift over time due to selection and drift processes. Thus, a change in genetic parameters can be observed over time, which leads to biased results of the breeding value estimation if it is not accounted for [3].

We therefore reevaluated the genetic parameters that were used for the BLUP breeding value estimation every five years. We did so based on the within-family variance, to avoid biased results due to the Bulmer effect [54]. Following ([3], Equation (7)), the variance of the Mendelian sampling, $\delta_{D}=\left[\begin{array}{c} \delta_{D}^{\mathrm{mat}}\\ \delta_{D}^{\mathrm{dir}} \end{array}\right]$, in the inheritance from a queen *Q* with inbreeding coefficient $F_{Q}$ to a drone *D* can be estimated as

$$\mathrm{var}\left(\delta_{D}\right)=\left(1-F_{Q}\right)\cdot \Sigma_{A}.$$

Drones are hereby interpreted as diploid but homozygous at all loci, as it is sometimes assumed in theoretical honeybee genetics [3,55]. In every fifth year, we let all breeding queens ($Q_{1},\ldots ,Q_{N}$) of that year produce 100 drones ($D_{Q_{i},1},\ldots ,D_{Q_{i},100}$) each. With

$$\delta_{D_{Q_{i},j}}:=\mathrm{TBV}\left(Q_{i}\right)-\mathrm{TBV}\left(D_{Q_{i},j}\right),$$

we then calculated the additive genetic variance in that year as

$${\hat{\Sigma }}_{A}=\frac{1}{100N-1}\sum_{i=1}^{N}\frac{1}{1-F_{Q_{i}}}\sum_{j=1}^{100}\delta_{D_{Q_{i},j}}\delta_{D_{Q_{i},j}}^{\prime }.$$

For this and the following four years, the breeding value estimation was performed with the use of the newly estimated ${\hat{\Sigma }}_{A}$. Although genetic parameters changed over time, BLUP was always performed with the full pedigree and all historical performance records, as is the current practice in the European breeding programs represented by beebreed.eu [53].

### 2.4. Analysis of Simulation Output

The analysis of genetic changes in the populations focused on three key values: genetic gain, genetic variance, and generational rate of inbreeding. While genetic gain is the main incentive for breeding, the development of genetic variance and inbreeding signify the sustainability of breeding programs. The genetic gain in a specified year was measured as the average sum of maternal breeding values of queens and direct breeding values of worker groups. This so-called *performance criterion* signifies the average phenotypic superiority over the base population [3]. Genetic variance was measured in the so-called *inheritance criterion*; i.e., we considered the variance of the sum of maternal and direct breeding values of queens. Since only queens are able to reproduce, this value reflects the population’s potential for further genetic change [42]. The generational inbreeding rate ΔF is defined as $\Delta F=\frac{F_{t+1}-F_{t}}{1-F_{t}}$, where $F_{t}$ and $F_{t+1}$ are average inbreeding coefficients in successive generations, and is usually assumed to be constant over generations. Consequently, we calculated ΔF as $\Delta F=1-{\left(1-F_{100}\right)}^{2.5/99}$, where $F_{100}$ is the average inbreeding coefficient of queens in year 100, 99 is the number of years elapsed since year 1, and 2.5 is the average generation interval (two years on the maternal path and three years on the paternal path). Individual inbreeding coefficients were calculated from the pedigree. All analyses were carried out using the statistical software R [56].

## 3. Results

### 3.1. Overview of the Simulation Outcome

**Figure 1:**
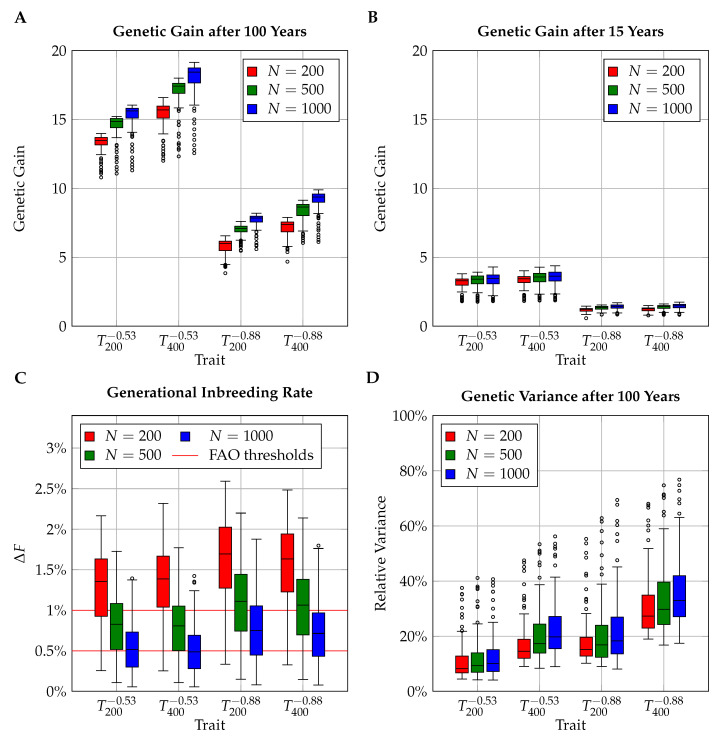
Simulated results for genetic gain after 100 (**A**) and after 15 years (**B**), generational inbreeding rates (**C**), and remaining genetic variance after 100 years (**D**). Individual boxplots are drawn for combinations of population sizes (N=200, N=500, N=1000) and selection traits ($T_{200}^{-0.53}$, $T_{400}^{-0.53}$, $T_{200}^{-0.88}$, $T_{400}^{-0.88}$), signifying different numbers of influential loci ($N_{l}=200,400$) and correlations between maternal and direct effects ($r_{md}=-0.53,-0.88$). The boxplots comprise the results obtained for varying sister group sizes ($1\leq k_{d}\leq 10$) and selection rates for sires ($1\leq k_{s}\leq 10$).

The genetic gain after 100 years varied between 10.80 and 19.15 units in simulations with $r_{md}=-0.53$ and between 3.85 and 9.90 units in simulations with $r_{md}=-0.88$. The 24 repetitions of the simulations revealed an average standard deviation in genetic gain after 100 years of 0.54 units. Genetic gain in population L (*N*=1000) was on average 0.76 units higher than in population M (N=500) and 2.09 units higher than in population S (N=200). Traits that were determined by 400 loci yielded on average 1.89 units higher genetic gain (see Figure 1A). The effects of population size and number of loci on genetic gain were only substantial in the long term; after 15 years there were only marginal differences (see Figure 1B). Generational inbreeding rates ranged between ΔF=0.06% and ΔF=2.59%. Larger populations showed lower inbreeding rates, while the number of loci with influence on the trait had no effect. The traits with strong negative correlation between queen and worker effects, $r_{md}=-0.88$, yielded higher generational inbreeding rates than the traits with moderate correlation, $r_{md}=-0.53$ (see Figure 1C). Comparing the generational inbreeding rates with the thresholds suggested by the FAO, we found that in the small population (N=200), 21.25% of all combinations of breeding schemes and traits resulted in a generational inbreeding rate ≤1% and 6% of the combinations yielded a generational inbreeding rate ≤0.5%. In the larger population, the corresponding percentages were 54.75% and 19% (N=500), and 82.5% and 39.75% (N=1000). The standard deviation of inbreeding rates over the 24 repetitions was 0.08%. When we compared the additive genetic population variance after 100 years with the variance in the base population, we found that on average, 21.74% of the initial variance was maintained (see Figure 1D). Among the schemes with generational inbreeding rates below 1%, the maintenance rate of genetic variance was 27.05% (35.49% for ΔF≤0.5%).

### 3.2. Comparison of Breeding Schemes

#### 3.2.1. Generational Inbreeding Rates

**Figure 2:**
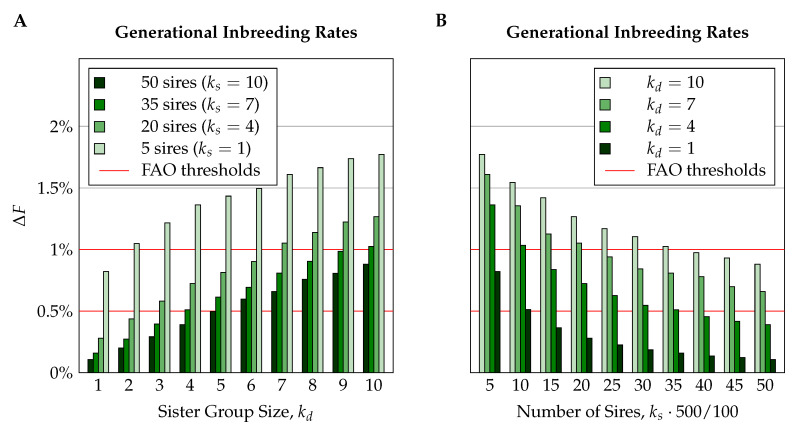
Generational inbreeding rates for different selection schemes on a population of N=500 colonies per year for trait $T_{400}^{-0.53}$ with moderate negative correlation between maternal and direct effects ($r_{md}=-0.53$), influenced by 400 loci. The subfigures show the influences of different sister group sizes (**A**) and numbers of sires (**B**).

In all populations and for all traits, schemes with sharper selection resulted in higher generational inbreeding rates; i.e., generational inbreeding rates increased with the sizes of queen sister groups, $k_{d}$, and decreased with higher numbers of sires (see Figure 2 for a typical example). While for large numbers of mating stations the generational inbreeding rates depended nearly linearly on the sizes of sister groups, the dependency was slightly sublinear for small numbers of mating stations. Depending on the number of mating stations, an increase of sister group sizes by one queen caused average increases in the generational inbreeding rate between 0.09% and 0.16% for N=200, between 0.09% and 0.15% for N=500, and between 0.06% and 0.13% for N=1000 (values in absolute percentage points). When it came to the influence of the number of sires, we found a convex dependency; i.e., when the total number of sires was low, the addition of further sires decreased the generational inbreeding rates more than when the number of sires was high. Stepping from $k_{s}=1$ to $k_{s}=2$ decreased the generational inbreeding rate on average by 0.29%, while the step from $k_{s}=9$ to $k_{s}=10$ only yielded an average decrease by 0.04% (absolute percent points).

#### 3.2.2. Sustainable Genetic Gain

**Figure 3:**
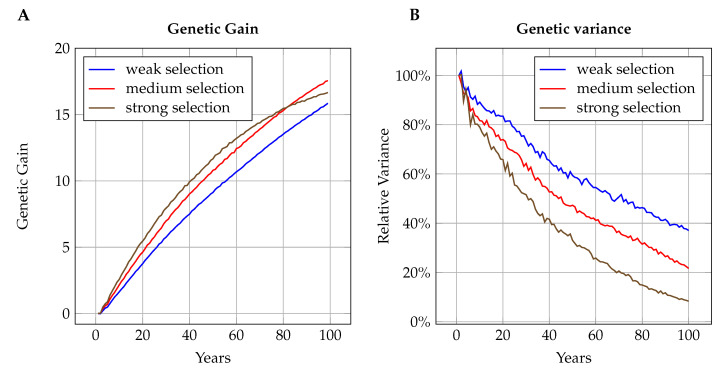
Development of genetic gain (**A**) and genetic variance (**B**) over time for three selection intensities: Strong selection ($k_{d}=9$, $k_{s}=1$), medium selection ($k_{d}=4$, $k_{s}=6$), and weak selection ($k_{d}=2$, $k_{s}=9$). Initially, intense selection yields higher rates of genetic gain, but the severe reduction of genetic variance leads to reduced gain in later years. Graphs are shown for a population of 500 queens per year and selection for trait $T_{400}^{-0.53}$ with 400 influential loci and moderate correlation between effects ($r_{md}=-0.53$).

**Figure 4:**
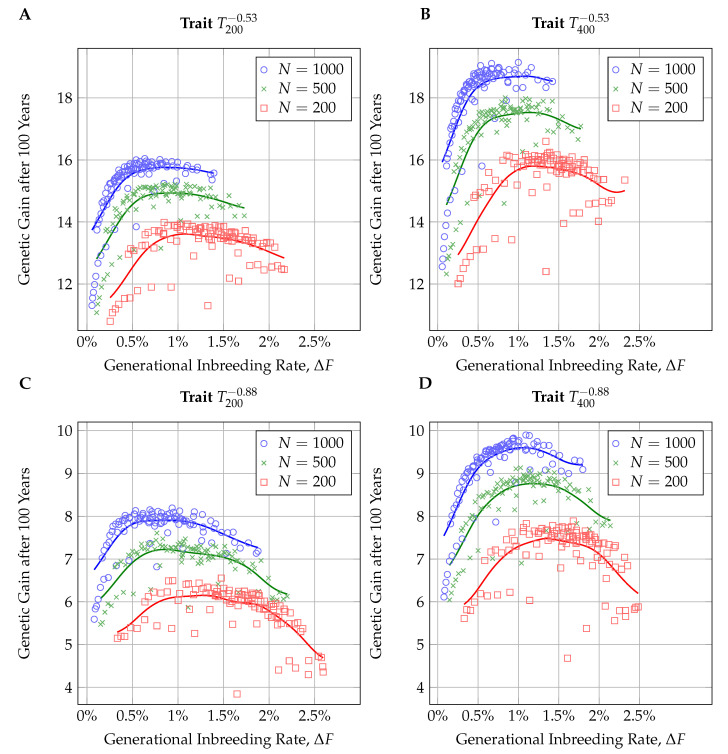
Scatter-plot of genetic response after 100 years vs. generational inbreeding rate. The regression curves were obtained using a Gaussian kernel with bandwidth 0.003, as they were implemented in the R package np [56,57]. Subfigures (**A**–**D**) represent the four simulated traits, defined by number of influential loci ($N_{l}=200,400$) and the correlation between maternal and direct effects ($r_{md}=-0.53,-0.88$).

Looking at the genetic gain after 100 years of selection, we observed two opposing effects influencing the outcomes. A strong selection led to high rates of genetic gain in the first years but also caused severe reduction of genetic variance which diminished the gain rates in later years (see Figure 3 for examples). As a result, when comparing accumulated genetic response after 100 years with generational inbreeding rates, we found inverted-U shaped connections in all of the simulations (see Figure 4). In larger populations, the maximum rates of genetic gain were reached at lower generational inbreeding rates than in smaller populations.

We considered a breeding scheme to be competitive in the 100 year frame if the genetic gain after 100 years was not more than 5% lower than in the breeding scheme with the highest genetic gain. Table 2 gives an overview of the competitive breeding schemes (CBSs) in the different scenarios. When there was a strong negative correlation between maternal and direct effects ($r_{md}=-0.88$), there were significantly fewer CBSs than for the moderate correlation ($r_{md}=-0.53$). Furthermore, larger populations allowed for more CBSs than smaller ones. All combinations of trait and population size allowed for CBSs with generational inbreeding rates below 1%. However, the stricter threshold of ΔF≤0.5% was only kept in the larger populations (N=500, N=1000) and mostly for the traits with moderate correlation between effects ($r_{md}=-0.53$).

In the small population (N=200), most breeding schemes which turned out both competitive and sustainable (ΔF≤1%) featured sister group sizes of $k_{d}=3$ or $k_{d}=4$. The number of mating stations in these breeding schemes was generally high; $k_{s}$ was mostly ≥6.

In population M (N=500), most sustainable CBSs had sister group sizes of $k_{d}\geq 4$ and the numbers of sires were mostly determined by values of $k_{s}\geq 4$. Stronger selection of dams, i.e., larger sister group sizes, required higher numbers of mating stations to remain sustainable. When the number of mating stations was very high ($k_{s}\geq 9$), the traits with medium correlation between effects ($r_{md}=-0.53$) even allowed for sustainable selection with the largest size of sister groups ($k_{d}=10$). For the other traits ($r_{md}=-0.88$), the maximum sister group size that still allowed for sustainable breeding was $k_{d}=7$, but only when there were many sires for the queens to mate with ($k_{s}\geq 9$). The few CBSs in population M with generational inbreeding rates below the stricter threshold of 0.5% mostly had sister group sizes of $k_{d}=4$ and large numbers of sires ($k_{s}\geq 8$).

In the large population (N=1000), 20.5% of all CBSs passed the strict sustainability criterion of ΔF≤0.5%. For these schemes, the sister group size $k_{d}$ mostly lay between 4 and 6 and the number of sires was determined by values of $k_{s}\geq 4$. Trait $T_{400}^{-0.88}$ only allowed for CBSs with the weaker sustainability criterion ΔF≤1%. The CBSs that fulfilled this criterion featured sister group sizes of five or more queens. Here, large numbers of sires required a sharp selection on the dam’s path in order to stay competitive. For $k_{s}=10$, only breeding schemes with sister group sizes of $k_{d}\geq 8$ were competitive.

All CBSs experienced severe losses of more than 63% in genetic variance over the course of 100 years. Losses were worst for trait $T_{200}^{-0.53}$, where even the competitive breeding schemes with the lowest generational inbreeding rates lost over 85% of the initial genetic variance in the population.

### 3.3. Short-Term Genetic Gain

**Figure 5:**
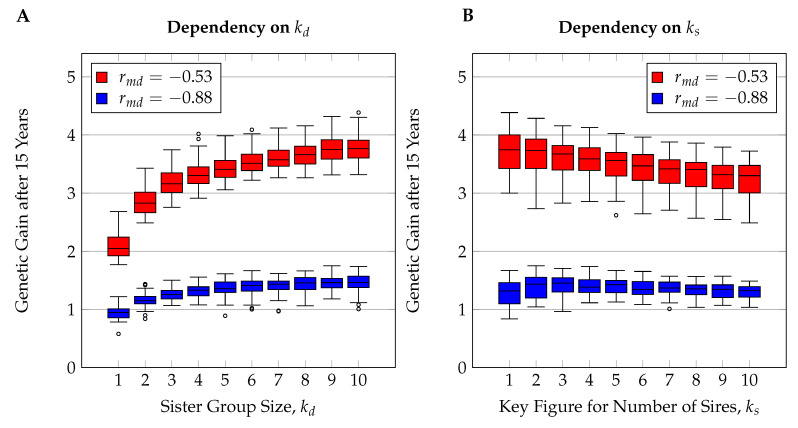
Box plots showing the average genetic gain after 15 years for different sister group sizes (**A**) and different numbers of sires (**B**). In part (**B**), values for sister group size $k_{d}=1$ are omitted to avoid an excess of outliers.

The simulated scope of 100 years lies far outside the planning range of practical beekeepers or animal breeders in general. To evaluate the breeding schemes for attractiveness for honeybee breeders, we therefore also investigated genetic gain after 15 years of breeding. Here the breeding success depended mostly on the breeding scheme and the genetic correlation between effects but not so much on the number of involved loci or the population size (see Figure 1B). We found that sharper selection on the dam’s path, i.e., larger sister group sizes, led to greater response to selection (see Figure 5A). A similar influence was observed for the number of sires in the traits with medium correlation between effects ($r_{md}=-0.53$). For the traits with strongly negative correlation ($r_{md}=-0.88$), however, there was no clear directional impact of the number of mating stations on the genetic gain after 15 years (Figure 5B).

The genetic gain after 15 years, dependent on the sister group size $k_{d}$ of breeding queens, followed the law of diminishing marginal utility; i.e., the larger the sister group size was, the smaller the additional genetic gain that was achieved by further increasing the sister group size. Compared to a sister group size of $k_{d}=10$, which on average yielded the highest genetic gain after 15 years, the genetic gain was reduced by 4.92% for $k_{d}=6$, by 10.03% for $k_{d}=4$, and by 14.17% for $k_{d}=3$.

The number of sires, represented by $k_{s}$, had a smaller effect on the genetic gain after 15 years than decisions on the dam selecting path. In the traits with moderate genetic correlation ($r_{md}=-0.53$), every increment of $k_{s}$ by one caused an average reduction in genetic gain by 0.92% in the small population S, by 1.64% in the medium population M, and by 2.03% in the large population L. When the genetic correlation was stronger ($r_{md}=-0.88$), the average genetic gain after 15 years was highest for $k_{s}=3$ with 1.39 units and ranged lowest for $k_{s}=10$ at 1.25 units (9.64% lower).

## 4. Discussion

### 4.1. Reality Check on Assumptions

Our simulation study suggests that sustainable breeding for honeybees is generally possible, even for populations consisting of as few as 200 colonies per year. However, as for any mathematical model of biological processes, our analysis is based on assumptions. In the following, we discuss the plausibility of the most crucial premises of this study.

#### 4.1.1. Population Structure

Our simulated populations had a very homogeneous structure. Every year comprised the same numbers of breeding colonies, selected queens, and mating stations. Furthermore, all queens were selected at constant rates for the same single trait and strict truncation selection was applied. Real populations are much more diverse. Because breeders are usually organized locally, a significant amount of genetic transfer occurs locally, leading to clustered populations with partly connected sub-populations which mix only to a limited extent. In comparison with the simulated homogeneous population, clustered populations have higher inbreeding rates and thus smaller effective population sizes [58]. In places with unfavorable environmental conditions, the population size may reduce drastically in some years, if only few colonies are overwintered successfully [59]. The occurrence of such population bottlenecks may further increase inbreeding rates [60]. However, there are also factors which reduce inbreeding in reality compared to our study. Most notably, the assumption of truncation selection across families is unlikely to be followed strictly. While offspring of the queens with the absolute highest estimated breeding values will certainly be coveted, some breeders will also select primarily among the offspring of their own queens. The result is likely to be a hybrid scheme between across-family and within-family selection. Similar selection rates generally yield lower rates of inbreeding when within-family selection is applied [61]; Moritz [46] explicitly confirmed this for the honeybee. Moreover, selection decisions are further diversified by different breeding foci of individual breeders, following a variety of trade-off decisions between more than one trait [62]. Furthermore, selection foci may shift over time because of environmental changes or technical innovations. Taking both parts into consideration, we conclude that while our simulation studies inevitably are imperfect reflections of real honeybee breeding, we do not have any evidence that our results are thereby biased in a specific direction.

#### 4.1.2. Genetic Model

Our simulations used a genetic model with a finite number of unlinked loci, as is recommended by Plate et al. [3] for long-term studies. The argument made for the finite locus model in this previous study is that in comparison with the infinitesimal model, it is a more accurate reflection of biological reality and that it minimizes the risk of underestimating the loss of genetic variance due to drift and selection. However, Hill [63] argues that finite locus models actually exaggerate the extent of decrease of genetic variance. Furthermore, we did not model any non-additive effects which may have a positive influence on the maintenance of genetic variance [64]. The severe losses we observed in this study might therefore draw an overly dramatic picture. Partly because of this reason, we decided to focus our analysis mainly on generational inbreeding rates rather than maintenance of genetic variance, because inbreeding rates depend less on the choice of the genetic model [3]. We included neither linkage nor mutations into our model. While linkage has been shown to be of little effect in the framework of this study [3], mutations could potentially help to retain genetic variance over the long period of 100 years [65].

#### 4.1.3. Analysis of Results

Our analyses relied on 24 repetitions of a Monte Carlo process. The standard deviations for the results in genetic gain and inbreeding were small in comparison to their absolute values; hence, we obtained stable results. This is in line with an earlier study [3] that showed that reliable results in breeding simulations for honeybees can be achieved with relatively few repetitions.

We based a large part of our analysis on the assumption that sustainable breeding is possible if the generational inbreeding rate is lower than 0.5% to 1% per generation, as is recommended by the FAO. These thresholds are mainly based on theoretical works by Franklin [66] and Soulé [67]. As pointed out by Shaffer [68], recommendations of this kind should be regarded as "rough guidelines rather than specific prescriptions." Because breeding depends on several random processes, an individual realization of a breeding scheme may produce higher inbreeding rates than predicted by our simulations. But since the 24 replications of our simulations revealed only small standard deviations of generational inbreeding rates (0.08%), this risk appears negligible. The FAO thresholds have occasionally been criticized as too high by conservation biologists [69,70], but are widely thought of as plausible in animal breeding [71]. For honeybees, it has been shown that inbreeding depression has negative influences on traits such as honey yield and swarming behavior [72,73]. However, these studies do not give reliable estimates of which inbreeding rates allow for sustainable breeding of honeybees. Compared to other species, in honeybees, there is an additional way in which inbreeding has negative effects on the colony; namely, the genetics at the sex-determining locus *csd*. Worker brood that is homozygous at this locus is removed from the hive, leaving gaps in the brood and thus acting negatively on the colony size [74]. Zayed [75] thus argues that honeybees are especially vulnerable to inbreeding and special care has to be taken. Contrary to this effect, inbreeding depression due to homozygosity of recessive deleterious alleles at other loci is likely to be expressed in haploid drones directly. The mating procedure may thus implicitly select against such alleles and alleviate consequences of inbreeding [76]. We are not aware of any dependable data that support a claim that generational inbreeding rates in honeybee breeding programs should obey different thresholds than those in selection programs of other livestock.

We introduced the notion of a competitive breeding scheme (CBS) to judge breeding decisions in a long-term frame. Breeding schemes were considered competitive if genetic gain after 100 years was reduced by at most 5% in comparison to the most successful scheme. On average, these 5% roughly represented the standard deviations obtained from the 24 repetitions. Therefore, this definition compensates for the fact that individual realizations of breeding schemes may vary in their results, while still allowing one to distinguish the most successful breeding schemes. We think of it as a valuable tool in the assessment of breeding schemes. However, it should only be used in combination with other criteria that provide a direct evaluation of inbreeding or genetic variance. Table 2 and Figure 4 show that, particularly in small populations, breeding schemes can be evaluated as "competitive" while accumulating high inbreeding rates.

The time point after 100 years to evaluate the long-term effects was to some extent an arbitrary choice. Figure 3 shows that even with intense selection, there is still some, albeit reduced genetic progress in the last years simulated. Had the evaluation time been chosen to be later, it is likely that fewer of the unsustainable breeding schemes would have appeared to be competitive. Under simplifying assumptions, Dempster [77] and Robertson [78] showed that under indefinite selection, the highest ultimate genetic gain is to be expected if about half of all individuals are selected as parents in each generation. This corresponds to lower selection rates than in any breeding scheme considered competitive in the present study.

### 4.2. Influences of Simulation Parameters

#### 4.2.1. Population Size

Population size proved to be a key factor that is to be considered in the design of breeding strategies. Larger populations proved superior in regard to genetic gain, conservation of genetic variance, and avoidance of inbreeding (Figure 1). Furthermore, relations between various influencing factors can be determined more clearly in larger populations; e.g., the connection between inbreeding and genetic gain in Figure 4 appears more scattered for the smaller populations. This is due in part to averaging effects leading to less variable simulation results in larger populations [3], and in part to the fact that inbreeding effects are more punishing in smaller populations since favorable alleles can be lost more easily.

The present simulations included population sizes of up to 1000 breeding queens per year. This is eight times smaller than the size of the Central European breeding population of *A. m. carnica* that is registered in beebreed.eu [53]. However, a single repetition of a simulation of a breeding scheme for a specific trait in the large population had a runtime of approximately nine hours on the high performance computing cluster of the North-German Supercomputing Alliance (HLRN) and the simulation time depended clearly superlinearly on the population size. The scope of the study presented here with 24 repetitions for 1200 settings was only possible due to massive parallelization efforts. Running simulations for populations of a size like that in beebreed.eu thus seems currently out of reach. The beebreed.eu population has strong regional relationship clusters, which, as described above, reduce its effective population size. Extrapolations from the simulated data are therefore difficult and prone to imprecision. But our simulations showed that larger population sizes are also more forgiving when it comes to breeding decisions, having higher total numbers of competitive breeding schemes. Therefore, we are confident that good breeding results can be achieved for populations of the size of Central European *A. m. carnica* if one follows a rough guideline that selection can be slightly more intense than in the simulated population L, consisting of 1000 colonies per year.

The simulations also do not cover very small populations of less than 200 queens per year. One can argue that sustainable breeding for such populations will require even lower sister group sizes than the smallest simulated population, S, and that a higher proportional number of sires will be necessary. However, as the effective population sizes for sustainable breeding should not drop below $N_{e}=50$ to $N_{e}=100$, these numbers also form strict lower bounds on the actual population size. Moritz [46] argues that breeding of very small populations should only be applied over few generations. In recent years, great advances have been achieved in cryopreservation methods for the honeybee [79], potentially opening the door for sustainable breeding for very small populations [80]. However, the use of preserved semen or embryos would lead to extended generation intervals, and therefore a slow-down of the genetic progress.

#### 4.2.2. Genetic Parameters

Negative correlations between effects complicated breeding efforts, leading to lower genetic gain and increased generational inbreeding rates (see Figure 1). Both effects have previously been observed in simulations on honeybees [3,43] and in more general settings of animal breeding [81,82]. The increased generational inbreeding rates are ultimately an effect of the reduced total heritability under negative correlation between effects [83]. Higher generational inbreeding rates in similar selection schemes resulted in a more pronounced inverted-U shape in the relation between inbreeding and genetic gain (Figure 4) and thus fewer competitive breeding schemes. Thus, when breeding is targeted at a trait for which a strong negative correlation between effects is known, sister group sizes should be reduced and/or the number of sires should be increased in comparison to traits with less pronounced correlations. Similar measures should be taken in cases wherein one knows that a trait is influenced by only few loci, because in this case, similar rates of inbreeding translate to more severe losses of genetic variance. In practice, however, we suspect it to be difficult to customize breeding schemes to the genetic parameters of selection traits. Especially in small populations, the estimation of genetic parameters is often difficult and likely to be imprecise [39,41]. Moreover, most breeding efforts include a variety of selection traits with different genetic parameters.

#### 4.2.3. Sister Group Sizes

By implementing sister group sizes between $k_{d}=1$ to $k_{d}=10$, we covered a wide range of selection rates on the dam’s path: between 10% and 100%. This signifies that there is great potential to shape selection schemes via this parameter. Our simulations suggest that in many settings for honeybee breeding it is a favorable decision in the long term to follow breeding schemes with sister group sizes $k_{d}$ between three and six queens (selection rates between 17% and 33%), while the ideal number rises with the population size. On this base, the official recommendations of sister group sizes of $k_{d}=6$ to $k_{d}=8$ [37,38] seem reasonable for the large Central European population of *A. m. carnica* but should not simply be transferred to smaller populations. Recommendations of large sister group sizes of twelve or more queens should be seen skeptically. However, one has to take into consideration that our simulations reflect populations that remain closed for many years. We can therefore give no clear suggestions for combination breeding schemes, such as that of Buckfast bees, which rely substantially on a steady influx of genetic material from various subspecies [84]. Pure-bred populations are not necessarily completely closed either; for an Austrian breeding population of *A. m. carnica*, managed by the bee breeders’ organization Biene Österreich, it has been reported that each year about a quarter of the colonies are newly introduced with an unregistered dam or sire [41]. Introducing unrelated colonies to the breeding population will decrease inbreeding rates but potentially hamper genetic gain. In the first years after bringing new colonies into the population it might thus be advisable to breed with larger sister group sizes to increase the accuracy of breeding value estimation [85]. If the breeding population can be enlarged early on through the addition of further colonies, this will in the long run have positive effects on the genetic gain [86]. However, for the most endangered subspecies, such as *A. m. ruttneri* and *A. m. siciliana*, the introduction of new colonies is not an option, because breeding population and total population coincide.

The use of smaller sister groups in smaller populations comes with a higher workload—because more colonies have to be prepared for queen rearing [5]—and smaller genetic gain in the short term (Figure 5). However, one may assume that beekeepers who are willing to work with endangered honeybee subspecies are committed to ideals beyond pure profit maximization. A 2010 study found a wide range of motivations of European cattle breeders to work with local breeds, including tradition, regional adaptation, and conservation efforts [87]. With a sister group size of $k_{d}=4$, one can expect to still reach almost 90% of the maximum genetic gain after 15 years; we are thus optimistic that breeders can be convinced to follow such sustainable schemes.

If local honeybee breeding proves successful, it is likely to attract more breeders and thus yield a growing breeding population [17]. The then larger breeding populations will enable more intense selection schemes in the future.

#### 4.2.4. Number of Sires

In our simulations, selection for dams of sires was generally stricter than selection for dams of breeding queens, which reflects reality in honeybee breeding. Limited availability and high maintenance costs for isolated mating stations are the main reasons for the stricter selection on the sires’ path in practice [5,88]. The selection rate for sires had a lower influence on the short-term genetic gain than the selection rate for dams (Figure 5). This is in line with previous simulation studies on honeybees [3,43].

For the traits with strongly negatively correlated maternal and direct effects, intensified selection rates for sires sometimes translated to lower selection intensities (see Figure 5B). The reason is that for these traits, the accuracy of estimated breeding values declines with the number of mating stations [43]. Regarding optimal numbers of mating stations, we observed that while the small population, S, required high relative numbers of sires for sustainable breeding (signified by large values for $k_{s}$), an oversupply of sires proved disadvantageous to long-term genetic gain in the large population, L. This is in line with the general fact that inbreeding rates, which we took as measurement for sustainability, depend mainly on the absolute numbers of selected parents. Based on numbers of sires in existing breeding populations, we do not see an abundance of sires as a realistic threat in practical breeding with large populations [53]. On the contrary, many breeding organisations for small populations will face difficulties to provide a sufficient number of mating stations, given the limited availability of geographic places with suitable conditions [88]. Therefore, alternatives to classical mating stations should be promoted, the most important of which is probably artificial insemination of queens [89]. When inseminating queens instrumentally, breeders can mimic the situation on mating stations; i.e., use the sperm of drones from a sister group of drone-producing colonies to fertilize the queens [5,7]. But a wide range of alternative procedures is possible, reaching from the use of mixed semen from large numbers of drones [90] to inseminations with drones from a single colony [91] or even single-drone inseminations [92]. In his simulation studies, Moritz [46] propagated artificial insemination with mixed sperm of many drones. In comparison to mating stations, these schemes yield higher genetic diversity within worker groups but not necessarily between individual queens. Furthermore, they do not allow to include paternal inheritance into the BLUP breeding value estimation and thus are not fully compatible with modern strategies of genetic evaluation. Ultimately, the long-term influences of different insemination practices on generational inbreeding rates, genetic gain, and genetic variance are yet to be investigated.

Whereas sister group sizes can easily be established by individual breeders, the design of the sires’ part of breeding schemes are mainly determined by breeding organizations. Burgeoning honeybee breeding efforts in Europe and potential breeding of *Apis cerana* in Asia can only be successful in the long run if breeding organizations manage to provide a sufficient number of mating stations and/or adequate training for instrumental insemination.

### 4.3. Further Breeding Schemes

The breeding schemes of Page and Laidlaw [47] build on several breeding lines that are kept separated for several generations and subsequent outcrossing. To this day, they are still sporadically in use [15,93]. However, we do not see them as adequate alternatives for long-term breeding because inbreeding in the individual lines will increase drastically and after outcrossing in a closed population, the queens of the next generation will be heterozygous but closely related, resulting in accelerated inbreeding in the next generations. Furthermore, one should remark that Page and Laidlaw based their analysis exclusively on considerations about the sex locus *csd*, which they assumed to have between 6 and 19 alleles in a population. In the meantime, it has been shown that this number is in reality at least one order of magnitude higher [94]. We therefore see across-family selection with appropriate selection rates, as presented in this study, as the more promising breeding alternative.

In the future, further improvements could be reached with more sophisticated selection schemes. Optimum contribution selection was designed to maximize genetic gain at a predefined generational inbreeding rate [95]. However, to this day, this concept has not been adjusted to the genetic peculiarities of the honeybee.

Furthermore, we see both potentials and risks in the emergence of genomic selection methods in the honeybee [96,97,98]. In practice, the introduction of genomic selection has led to increased inbreeding rates and loss of genetic variance in cattle populations [99,100]. However, it has been proven that genomic breeding can outperform traditional methods in respect to maintenance of genetic variance and effective population size when applied correctly [101,102,103].

## 5. Conclusions

Sustainable across-family selection of honeybees can be achieved for breeding populations with at least 200 colonies per year. In populations with 200 colonies, sister group sizes should not exceed three to four queens in order to avoid high inbreeding rates. For larger populations (500 to 1000 queens per year), this number can be increased to four to six queens, if a sufficient number of sires can be provided. The number of sires should generally be high, especially in small populations. Breeding schemes that are sustainable in the long term (100 years) may come at the cost of slightly decreased genetic gain in the short term (15 years).

The results we obtained here in silico have to be compared to in situ experiences that can be collected over the years and regulations should be adjusted accordingly.

## Figures and Tables

**Table 1:** Variable definitions.

Variable	Definition	Values
*N*	Number of breeding queens per year.	200,500,1000
$k_{d}$	Sister group size, number of queens produced by one dam.	1,2,…,10
$k_{s}$	Percentage of breeding queens selected to produce sires.	1,2,…,10
$S_{k_{s}}^{k_{d}}$	Selection scheme defined by key values $k_{d}$ and $k_{s}$.
$N_{l}$	Number of loci to determine a trait.	200,400
$\left(\sigma_{A,m}^{2},\sigma_{A,d}^{2}\right)$	Maternal and direct additive genetic variance.	(1,2)
$\sigma_{A,md}$	Genetic covariance between maternal and direct effects.	−0.75^a^, −1.25^b^
$r_{md}$	Genetic correlation between maternal and direct effects.	−0.53^a^, −0.88^b^
$\sigma_{E}^{2}$	Residual variance.	1
$\sigma_{A,\mathrm{IC}}$	Genetic standard deviation, inheritance criterion.	1.22^a^, 0.71^b^
$h^{2}$	Total heritability.	0.79^a^, 0.36^b^
$\left(h_{m}^{2},h_{d}^{2}\right)$	Maternal and direct heritabilities.	(0.53,0.34)^a^, (0.72,0.46)^b^
$T_{N_{l}}^{r_{md}}$	Trait determined by $N_{l}$ loci and correlation $r_{md}$ between effects.	$T_{200}^{-0.53}$, $T_{400}^{-0.53}$, $T_{200}^{-0.88}$, $T_{400}^{-0.88}$

^a,b^ Values with the same superscript only occurred in combination; the other values could be combined freely. Values for the traits (last row) result directly from the values of $N_{l}$ and $r_{md}$.

**Table 2:** Competitive breeding schemes (CBSs) after 100 years.

					CBS with Lowest ΔF				CBS with Highest ΔF			
		${N_{\mathrm{CBS}\;}}^{a}$	${N_{1\%}\;}^{b}$	${N_{0.5\%}\;}^{b}$	$k_{d}$	$k_{s}$	ΔF	var ^c^	$k_{d}$	$k_{s}$	ΔF	var ^c^
N=200	$T_{200}^{-0.53}$	70	14	0	3	10	0.64%	16.89%	10	3	2.01%	6.34%
	$T_{400}^{-0.53}$	48	5	0	4	10	0.81%	21.67%	10	5	1.82%	12.80%
	$T_{200}^{-0.88}$	16	3	0	2	6	0.88%	28.26%	8	8	1.73%	10.24%
	$T_{400}^{-0.88}$	37	1	0	3	9	0.92%	36.76%	7	4	1.94%	15.80%
N=500	$T_{200}^{-0.53}$	74	46	5	3	7	0.39%	15.45%	9	1	1.67%	5.29%
	$T_{400}^{-0.53}$	64	42	2	4	8	0.46%	25.10%	10	2	1.55%	12.59%
	$T_{200}^{-0.88}$	32	17	0	4	10	0.60%	27.34%	9	5	1.51%	8.49%
	$T_{400}^{-0.88}$	47	11	0	5	9	0.74%	37.22%	9	3	1.64%	15.66%
N=1000	$T_{200}^{-0.53}$	73	64	23	4	9	0.25%	15.13%	10	1	1.39%	5.15%
	$T_{400}^{-0.53}$	64	56	18	5	8	0.32%	25.06%	10	1	1.42%	11.02%
	$T_{200}^{-0.88}$	63	48	10	3	8	0.32%	34.02%	10	3	1.41%	7.29%
	$T_{400}^{-0.88}$	49	34	0	6	8	0.58%	35.46%	10	2	1.51%	13.35%

^a^ Number of competitive breeding schemes. ^b^ Number of competitive breeding schemes with generational inbreeding rate ΔF≤1%, resp. ΔF≤0.5%. ^c^ Genetic variance in year 100 relative to the genetic variance in the base population.

## Abbreviations

The following abbreviations are used in this manuscript:

BLUP	Best Linear Unbiased Prediction
CBS	Competitive Breeding Scheme
FAO	Food and Agriculture Organization of the United Nations
TBV	True Breeding Value
